# A Direct Entropic Approach to the Thermal Balance of Spontaneous Chemical Reactions

**DOI:** 10.3390/e26060450

**Published:** 2024-05-26

**Authors:** Michele D’Anna, Paolo Lubini, Hans U. Fuchs, Federico Corni

**Affiliations:** 1 Liceo Cantonale Locarno, 6600 Locarno, Switzerland; mbv.danna@gmail.com; 2 Liceo Cantonale Lugano 2, 6942 Savosa, Switzerland; paolo.lubini@edu.ti.ch; 3 Center for Narrative in Science, 8400 Winterthur, Switzerland; hans.fuchs@narrativescience.org; 4 Faculty of Education, Free University of Bolzano, 39042 Bressanone, Italy

**Keywords:** chemical potential, spontaneity of a reaction, dissipative processes, entropy and entropy production, molar entropy, entropy balance, generalized energy balance, exothermic and endothermic reactions

## Abstract

When working with, and learning about, the thermal balance of a chemical reaction, we need to consider two overlapping but conceptually distinct aspects: one relates to the process of reallocating entropy between reactants and products (because of different specific entropies of the new substances compared to those of the old), and the other to dissipative processes. Together, they determine how much entropy is exchanged between the chemicals and their environment (i.e., in heating and cooling). By making explicit use of (a) the two conjugate pairs chemical amount (i.e., amount of substance) and chemical potential, and entropy and temperature, respectively, (b) the laws of balance of amount of substance on the one hand and entropy on the other, and (c) a generalized approach to the energy principle, it is possible to create both imaginative and formal conceptual tools for modeling thermal balances associated with chemical transformations in general and exothermic and endothermic reactions in particular. In this paper, we outline the concepts and relations needed for a direct approach to chemical and thermal dynamics, create a model of exothermic and endothermic reactions, including numerical examples, and discuss how to relate the direct entropic approach to traditional models of these phenomena.

## 1. Introduction

In this paper, we outline a path to modeling the thermal effects of chemical reactions that makes direct use of experiential primitives for demonstrating the origins of the *spontaneity of a reaction* and of its *“heat” balance*. The primitives of models where the only coupling occurring is between chemical and thermal processes are *chemical potential* and *amount of substance*, and *temperature* and *entropy*, respectively.

Previously, we have formulated a direct entropic approach to both dynamical and steady-state models of thermoelectric devices [1]. Here, we take the same route to the thermal aspects of models of chemical reactions such as combustion and endothermic processes [2]. Such models use abstractions that are based upon and formed quite directly in experiencing dynamical causative phenomena, rather than applying formal constructs obtained indirectly from concepts in traditional equilibrium theories of chemical and thermal processes. Taking such an experientially natural approach [3] is assumed to lead to “economies” of learning and understanding, and to allow for easy access to Second Law analyses in *finite-time thermodynamics* (Chapter 9 in [2]; and [4,5,6,7]). This should be most useful to learners in introductory physics and chemistry courses, and to practitioners in fields such as chemical and energy engineering. Moreover, if we can create experientially natural forms of speaking about exothermic and endothermic chemical transformations, young learners and laypersons alike will profit.

The most basic concepts abstracted in experiencing thermal, electrical, chemical, and other phenomena are (a) *intensity* and its differences, i.e., potential differences (such as thermal and electrical tensions) that serve, imaginatively speaking, as “drives” for processes; (b) *extension*, i.e., quantity or amount of some “fluidlike stuff” (p. 9, [2]) for which laws of balance can be formulated (i.e., this “stuff” can be stored in physical systems, can flow and, in some cases, can be produced and/or destroyed); and (c) the *power* of processes upon which we can construct a generalized energy principle (Sections 2 and 4 in [3]). Examples of the first group of concepts are temperature, pressure, speed, electric potential, and chemical potential, along with their differences. In the second group, we find entropy, (fluid) volume, momentum, charge, and amount of substance, along with their flows and (in some cases) production and destruction rates. Finally, power and energy are the same for all phenomena. We shall call the concepts formed in this manner the *primitives* of our models and theories. Importantly, the same basic relation holds between potential differences, flows (or production/destruction rates), and the power of a process, irrespective of its type.

### 1.1. Foundations

We find the same abstractions underlying the formulation of the *theories of continuum physics* [2,8,9,10,11,12]. Other quantities are defined based upon the primitive concepts, and laws are expressed with their help. Second, there are the fundamental laws of balance of the quantities that are exchanged in processes, such as momentum, charge, or amount of substance; we call these quantities fluidlike. Third, we need special laws governing the behavior of, or distinguishing between, different bodies; these laws are called constitutive relations. Finally, we need a means of relating different types of physical phenomena. The tool that permits us to do this is energy. We use the energy principle, i.e., the law that expresses our belief that there is a conserved quantity that appears in all phenomena, and which has a particular relationship with each of the types of processes (p. 9, [2]). The Gibbs Fundamental Forms, which are used as starting points of models in Gibbs’s thermodynamics, are derived from the foundational concepts used in continuum physics (CP) when applied to concrete systems and materials—they do not have the same experiential status as the primitives and basic relations upon which CP is founded (for examples of such derivations, see (pp. 221, 236, and Section 8.3 in [2])).

An important aspect of theories of systems and processes in CP is how they make analogous formal structures visible. High degrees of structure mapping [13,14,15,16] apply to laws of balance (“continuity equations”); the relations between potential differences, conductive flows, and power of processes; and constitutive relations such as capacitive laws and flow relations for both conductive and convective transports. Moreover, we can apply the ideas underlying CP to models of *spatially uniform dynamical systems* (UDS) and thereby produce a mathematically simpler subset of theories suited for introductory courses (on spatially uniform models, see (pp. 10–13, and Chapters 1–10 in [2]), and below in Section 2.1). Examples of models of chemical processes in UDS can be found in [2,17,18,19].

To return to the issue of experientially grounded abstractions vs. formal constructs obtained indirectly from the more basic concepts, let us take a brief look at thermodynamics. In a CP theory of thermal systems and processes, we use temperature and entropy as primitives and formulate the relation between temperature difference (thermal tension), flow of entropy through this temperature difference, and thermal power using Carnot’s [20] analogy between waterfalls and the fall of caloric (entropy) in heat engines [2,3]. A model of the coupling of chemical and thermal processes follows directly from this imagery—this is what we will show here, first qualitatively in this Introduction, and then formally in the rest of this paper.

If thermodynamics is based primarily on the First Law and related concepts, as is common in traditional equilibrium thermodynamics, important tools needed for understanding and direct modeling are missing. For this reason, auxiliary quantities based upon energy must first be derived [21,22,23], for which imagery is not available—apart from having to formally introduce the tools that may then be applied, we cannot render descriptions of systems and processes in simple natural language (and associated visual representations; see the qualitative example discussed below).

Readers of this paper will be familiar with what this means. The auxiliary formal tools derived from quantities related to energy are the *thermodynamic potentials*, such as enthalpy, free energy, and Gibbs free energy. For good measure, they involve entropy, for which figurative understanding is altogether missing if we follow the tradition. Then, add to these formulas the notions of exergy and anergy used in energy engineering. We contend that learners will be confused [24,25,26,27,28,29,30,31,32], and practitioners will be left to applying formal relations derived from equilibrium theories—which will restrict the range of applications found in finite-time thermodynamics and CP [2,4,5,6,7,8,9,10,11,12]—without the benefit of deeper understanding [3]. 

### 1.2. Qualitative Description of an Exothermic Reaction

**Figure 1:**
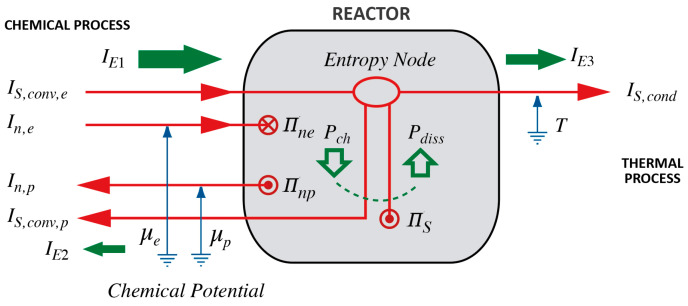
Process diagram depicting a spontaneous and purely dissipative chemical reaction running at steady-state. The diagram shows the model of how the thermal process is coupled to the chemical reaction. Note the thermal (entropy) balance expressed by flows and a production rate joining in the “Entropy Node”. For details on and examples of process diagrams, see [2].

Here is a case for which we produce an example of linguistic and graphical representations for a model in UDS. This demonstrates how the primitives can be used for constructing direct access to phenomena that might be difficult to understand if we were to use formally derived auxiliary concepts such as the traditional thermodynamic potentials. Consider burning a fuel such as methane in a furnace and, for simplicity’s sake, assume that everything runs at constant rates (steady-state); this leaves all quantities and relations constant in time. A description based upon an experientially natural approach uses figurative language that can be rendered as a sketch using visual equivalents of the conceptual metaphors appearing in natural language; this is what we have done in the process diagram in Figure 1. If you leave out notes and the reference to mathematical symbols in the description below, you essentially have a natural language “narrative” of what is happening in a (steady-state) combustion process used for heating purposes.

We observe a fuel (methane) and oxygen flowing into the furnace (educts e). At the same time, reaction products (such as carbon dioxide and water vapor; products p) and heat (S) leave the burner. Obviously, a lot of heat is being produced. Nothing else happens; there are no electrical or mechanical processes powered by the combustion of the fuel, and quantities such as temperature and pressure remain constant. [Note that the linguistic description creates an analogy between flows of fuels and other chemicals and heat; obviously, what we colloquially call “heat” is the entropy of a dynamical theory of heat [1,2,3]—from now on we shall use the technical term *entropy* for the experiential concept of *amount of heat*.]

[In a visualization of what we have just said, we would render all flows using the same visual metaphor, i.e., arrows symbolizing inflows and outflows of “stuff”. See the red lines and arrows leading into and out of the rectangle symbolizing the furnace in Figure 1, denoting a flow by $I_{X}$, where X stands for some fluidlike stuff, is the uniform dynamical systems equivalent of the mathematical symbols used in CP, and is equivalent to the practice found in lumped-parameter modeling of electric circuits. The flows expressly mentioned in the paragraph above are inflows of amount of substance ($I_{n,e}$), outflows of amount of substance ($I_{n,p}$), and entropy ($I_{S,cond}$).]

We know that the fuel and oxygen are consumed (at a rate $\Pi_{n,e}$) and reaction products are produced (at a rate $\Pi_{n,p}$). Destruction and production must happen at rates that equal the flows of the substances involved for a steady-state to hold. [In a visual representation, destruction and production rates are shown as sink and source symbols. Π is the UDS equivalent of symbols used in CP for production and destruction rates.]

The combustion of methane is spontaneous; experiencing spontaneous processes gives rise to the idea that in chemical transformations there must exist a chemical drive (tension) between educts and products (p. 251, [2]; p. 108, [33]), just as differences of hot and cold are experienced as thermal tensions, i.e., thermal drives [3]: educts enter at a chemical level (chemical potential) that is higher than the level at which products leave. In general, experience tells us that processes either use or establish drives. An existing drive allows for a spontaneous process, and in coupling to other processes the energy made available establishes new drives, i.e., tensions (see below; on making energy available, or *availability*, see [2,3,34,35]). [In Figure 1, the notion of level (i.e., potential) is represented metaphorically by introducing vertical (blue) arrows like those used for denoting “ground” in electrical circuit diagrams.]

It is obvious that a lot of entropy is leaving the furnace; so, where does it come from? Clearly, the chemicals flowing into and out of the reactor will transport some entropy ($I_{S,conv,e}$ and $I_{S,conv,p}$), but whatever the difference of these flows, huge amounts must have been produced in the furnace (at a rate $\Pi_{S}$); otherwise, we could not use the combustion of methane for heating purposes ($I_{S,cond}$). The sum of inflows and outflows of amounts of entropy and the rate at which it is produced must balance.

Finally, how is it possible to produce entropy, which is clearly a non-spontaneous process requiring energy? Chemicals undergoing spontaneous reactions are powerful relative to their products; this means that the spontaneous combustion of methane will make energy available at a certain rate, which we call *chemical power* ($P_{ch}$). This quantity is equal to the difference of the flows of energy carried by inflowing and outflowing substances ($I_{E1}$ and $I_{E2}$). Since there is only a single process powered by the reaction, all of the energy made available is used (at the rate $P_{diss}$) for driving the production of entropy (at the rate $\Pi_{S}$). Finally, the entropy leaving the furnace in the process we feel as “heating” ($I_{S,cond}$) will take a proper amount of energy with it ($I_{E3}$) so that all flows of energy will balance as well. [In the process diagram, we have represented energy flows as green fat horizontal arrows and rates at which energy is made available or used by green fat vertical arrows inside the reactor. Note that what is represented visually inside the symbol for the reactor is drawn in a manner suggestive of the interaction of chemical and thermal processes.]

In sum, the following are the main points that we learn: (1) the *difference of chemical potentials*, i.e., *chemical drive*, explains the idea of *spontaneity* of chemical reactions running “downhill” (see [2,33]); (2) *entropy* gives us a direct understanding of the *thermal balance* of such reactions [2]; and (3) *energy is made available by spontaneous processes* and then *used for driving caused processes*. Moreover, we do not need the full formal apparatus of CP for describing and modeling cases such as the one discussed here. As far as formalisms go, models of uniform (dynamical) systems (UDS) suffice, and the abstractions applied in these models are amenable to natural language “narratives”. Natural language becomes a powerful tool for understanding and modeling. In turn, the modeling process will profit from the conceptual metaphors [14,16,36] that can be rendered in visual form, as in process diagrams such as that in Figure 1 (for an application of process diagrams in a theory of control engineering, see [37]).

### 1.3. The Model to Be Discussed in This Paper: Assumptions and Limitations

After formalizing elements of the conceptual background in Section 2, we will construct the relations for a model of exothermic and endothermic reactions along the lines of what we sketched in the process diagram above in Figure 1 (Section 3); however, we will treat the general dynamical case, allowing equally for flow reactors and batch processes. We shall assume that the only processes occurring are chemical and thermal: all the energy made available by a spontaneous reaction will be used to produce entropy (i.e., we assume a *completely dissipative coupling of chemicals and heat*). 

However, when we interpret our general result for the entropy current in heating/cooling (which tells us if a reaction is either exothermic or endothermic) and present numerical examples, we assume *steady-state operation*, as we have done in the qualitative example described above. This requires an open flow system where reactants enter and products are removed. In this much simpler case—where we do not have to worry about temporal change—all variables (flows, including energy flows, rates of production and destruction, power, and temperature and pressure) will be constant. As in the general case, the main result of the modeling process will be a relation for the heating, i.e., the entropy flowing between the reactor and the environment ($I_{S,cond}$), in terms of the reaction rate, entropy carried by inflows and outflows of substances, and the relevant potentials (chemical potentials and temperature)—see Equation (18) below. The relation demonstrates that there are three possible cases of exothermic and endothermic processes.

Numerical examples for each of the three possible cases of exothermic and endothermic reactions will be presented in Section 4. Furthermore, in Section 5.2, we shall sketch how the traditional energy-based quantities (the thermodynamic potentials) can be derived from our direct approach to chemical and thermal dynamics. 

### 1.4. Teaching Thermodynamics and Thermochemistry

Over many years, the authors have taught introductory and applied courses in physics and chemistry to engineering students, students of primary education, and in AP courses in high school. During these years, in which we have developed a generalized approach to dynamical physical and chemical processes with a focus on fundamentals, applications, and the integration of experimenting and dynamical modeling [2,14,17,18,19,36], issues relating to learning have always been particularly relevant to us. We do not need to discuss the grave difficulties caused by formalisms removed from the very basic abstractions that constitute human understanding—the state of learning in traditional approaches to thermochemistry has been amply documented [24,25,26,27,28,29,30,31,32]. In this paper, we demonstrate how, in contrast, the abstractions formed quite directly in experience can be applied to models of thermochemical phenomena and, thus, create a foundation for a cognitively natural approach to learning about this fascinating field. In Section 5, we discuss how the teaching of physical chemistry can profit from a direct approach to the primitive concepts of chemical and thermal phenomena. A brief summary of our conclusions is presented in Section 6.

## 2. Conceptual Background

In this section, we shall collect the conceptual elements needed for explicit modeling and easy imaginative understanding of the *entropy balance* of a *spontaneous chemical reaction*. Conceptual simplification is achieved by a direct imaginative approach to the fundamental concepts of chemical and thermal processes—i.e., chemical potential and amount of substance, and temperature and entropy, respectively—and their relation to the generalized energy principle [2,3,18,19,33]. The basic assumptions concern (a) laws of balance for fluidlike quantities, (b) the relations that let us express chemical and thermal power, and (c) the expressions for energy currents in *conductive* transports [2,38]. The special (constitutive) relations concern the stoichiometry of a reaction and the expression for the *convective* transport of entropy in flows of reactants and products (Chapter 8 in [2]).

### 2.1. Spatially Uniform Processes and Consequences of Steady-State Operation

As suggested by Figure 1, we restrict our model to spatially uniform processes. Spatially uniform (dynamical) models are ubiquitous in science and engineering [1,2,3,4,5,6,7,17,18,37,39,40]. Simply put, in a spatially uniform system, variables do not depend upon spatial coordinates, in contrast to what is commonly assumed to be the case in spatially continuous models and theories of continuum physics [8,9,10,11,12]. In UDS, models are described by initial value problems in ordinary differential equations.

If we create a spatially uniform model of a single physical object, there will be only a single instance for each of the variables needed for a complete description of the system and the processes it undergoes, such as pressure, temperature, entropy, amount of substance, flows, production rates, etc. Put differently, quantities are functions of time only, and not of space, such as $p\left(t\right)$, $T\left(t\right)$, $S\left(t\right)$, $n\left(t\right)$, $I_{n}\left(t\right)$, $\Pi_{S}\left(t\right)$, etc. More generally, we can compose a more complex system of more than a single spatially uniform element—this is the case in lumped-parameter models that are widely used in electrical circuit models (see, for instance, [41]). In this case, there are separate instances of variables assumed to be uniform in each of the elements. In this paper, we treat the reactor as a single spatially uniform element (Figure 1).

As discussed in the Introduction, we shall limit our considerations to *steady-state* operation when interpreting certain results (Section 3.2) and presenting numerical examples (Section 4). Formally, there are two consequences of the assumption of steady-state operation: the rates of change of amount of substance and entropy, and of energy, will be equal to zero, i.e., dX/dt=0; and all flows, production and destruction rates, and potentials will be independent of time, as will power and energy flows.

If the processes considered are running at steady-state, our task of thinking about and explaining a situation using natural language will be simpler. In particular, we do not have to deal with initial value problems—a model reduces to algebraic relations. Nevertheless, our understanding of spontaneity of a reaction and its thermal balance will remain generally valid, independent of the particularities of a situation.

### 2.2. The General Balances of Amount of Substance, Entropy, and Energy

We now proceed to formalize concepts and relations for systems and processes of the type observed in the example presented in the Introduction. This will be carried out for the general case of a *dynamical* open (flow) system; steady-state and batch processes will be limiting cases of the general model. We shall restrict the discussion to generalized balances of amount of substance, entropy, and energy, and the relation between processes and power [2].

The extensive quantities of chemical processes are the amounts of substance ($n_{i}$) for the various chemical species (*i*) involved. For each of the substances, a generalized law of balance can be formulated:

(1) $$dn_{i}/dt=I_{ni,tot}+\Pi_{ni}.$$

**Figure 2:**
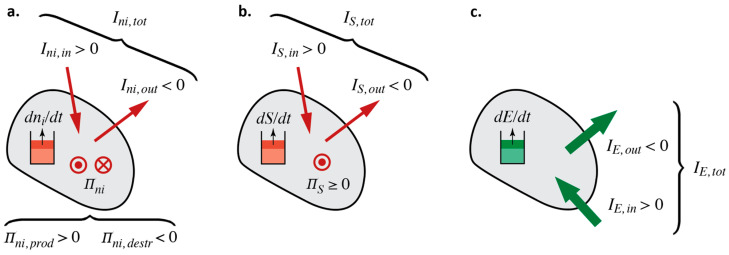
Schematic representation of the various elements—flows, production and destruction rates, and rates of change of stored quantity—used for modeling laws of balance: (**a**) amount of substance of species i ($n_{i}$)—if there are *k* different substances, there will be *k* equations of balance; (**b**) entropy (S); (**c**) energy (E).

Here, $dn_{i}/dt$ is the rate of change of amount of species i present in a storage element, $I_{ni,tot}$ is the sum of all currents of amount of substance of species i, and $\Pi_{ni}$ denotes the production/destruction rate of the same species (see Figure 2a). When a current $I_{X}$ in a law of balance denotes an inflow to a storage element, it will be taken as being positive; otherwise, if it denotes an outflow, it will be a negative quantity. Equivalently, $\Pi_{ni}>0$ denotes that a species is produced, whereas $\Pi_{ni}<0$ stands for its destruction. 

In general, it will be possible for a given species to flow both into and out of a reactor, and to be both produced and destroyed. Even though the following formalism will be kept general, for simplicity, it helps to think in terms of reactants (educts: e) and products (p)—reactants can only flow in and be consumed, whereas products can only be produced and flow out. 

As for entropy, this quantity can be stored, flow into and out of a physical object, and be produced (in irreversible processes) inside the object. Importantly, entropy can flow conductively and be transported by convection. Using symbols for rate of change of stored amount (dS/dt), conductive flows ($I_{S,cond}$), convective flows ($I_{S,conv}$), and production rate ($\Pi_{S}$), the overall dynamical law of balance is

(2) $$dS/dt=I_{S,cond,tot}+I_{S,conv,tot}+\Pi_{S}$$

(see Figure 2b). Note that $\Pi_{S}\geq 0$, i.e., it is strictly non-negative. Entropy can only be produced but never destroyed. 

Finally, energy obeys the generic form of a law of balance for a conserved quantity:

(3) $$dE/dt=I_{E,tot}$$

(see Figure 2c). Energy is conserved, i.e., there are no production or destruction rates. A change of the energy of a system (i.e., the energy stored in the system) must always be accompanied by energy transfer.

### 2.3. Stoichiometry, Individual Production/Destruction Rates, and Overall Reaction Rate

There is an important relation between amounts of the different species involved in a reaction. Production and destruction rates of amount of substance of reactants and products involved in a reaction are related by the rules of stoichiometry. We can write a reaction equation as follows:

(4) $$\nu_{A1}A1+\nu_{A2}A2+\cdots ⟶\nu_{B1}B1+\nu_{B2}B2+\cdots ,$$

where A1, A2 … represent the educts (reactants) and B1, B2 … the products; furthermore, $\nu_{Ai}<0$ and $\nu_{Bi}>0$. The *overall reaction rate* $\Pi_{nR}$ is related to each of the production/destruction rates $\Pi_{ni}$ of the individual chemical species by their respective stoichiometric number $\nu_{i}$:

(5) $$\Pi_{ni}=\nu_{i}\Pi_{nR}$$

For the signs of individual production/destruction rates to be correct, we need to assume that $\Pi_{nR}>0$. 

### 2.4. The Entropy of Substances and Convective Entropy Currents

The total entropy current in the law of balance given in Equation (2) can result from both *conductive* and *convective* transports. Conductive flows will be related to temperature differences, whereas convective currents depend upon how much entropy is contained in the flowing substances (which take their entropy along with them) and upon the current of a given substance. When we study the thermal balance of a reaction where educts and products flow into and out of a reactor, we need to know how to express the *convective entropy currents*. Essentially, a convective flow of quantities such as entropy, charge, and momentum equals the product of the (volume) density of this quantity and the (volume) current of the fluid material taking the quantity along. If we measure the current of a chemical taking entropy along in terms of amount of substance (rather than volume), i.e., if the current is $I_{ni}$, then the *convective current of entropy* related to this transport is given by

(6) $$I_{S,conv,i}={\hat{s}}_{i}I_{ni},$$

where ${\hat{s}}_{i}$ is the (absolute) molar entropy of species i, which is the amount of entropy found in a mole of that substance whose pressure and temperature are p and T, respectively. In general, ${\hat{s}}_{i}$ will depend upon these values: ${{\hat{s}}_{i}=\hat{s}}_{i}\left(p,T\right)$—we can say that ${\hat{s}}_{i}$ is the amount of entropy needed by a substance to be at some value of p and T, respectively.

Since different substances have different demands for entropy, the convective entropy current of a bundle of chemical substances (each having its own current of amount of substance $I_{ni}$) will be expressed as follows:

(7) $$I_{S,conv,tot}=\sum {\hat{s}}_{i}I_{ni}.$$

Therefore, in the case of a reaction where reactants and products are flowing into and out of a reactor, we will have to consider the different convective entropy currents associated with inputs and outputs (see Section 3).

### 2.5. Power and Energy Currents of Chemical and Thermal Processes

One of the most important ideas helping us understand processes in general, and interactions of different types of processes in particular, is found in the relation between processes and the rates at which energy is made available or used, i.e., their power. The expression for the power of a process derives from Carnot’s analogy [20] between heat (entropy) flowing from the furnace at $T_{1}$ to the cooler at $T_{2}$ and the fall of a current of water from height 1 to height 2 [for a modern rendering of this idea, see [2,3]].

Since the gravitational power of a waterfall equals $P_{grav}=∆\varphi_{grav}I_{m}$, by analogy, we can express the power of a fall of entropy by

(8) $$P_{th}=∆TI_{S},$$

where $I_{X}$ denotes the strength of a flow of a fluidlike quantity through a potential difference, which we always give a positive value. As we have stated above, this relation applies to all conductive processes, be they hydraulic, electrical, thermal, chemical, gravitational, or mechanical. It allows us to derive the expression for a current of energy carried by a conductive current of a fluidlike quantity. In the case of conductive flows of entropy, we have [2,15,42,43]

(9) $$I_{E,th}=T\;I_{S,cond},$$

and for the energy carried by a flow of chemical species i, by analogy, we have

(10) $$I_{E,ch,i}=\mu_{i}\;I_{ni},$$

where $\mu_{i}$ is the chemical potential of species i (see [2,33]).

Accepting these ideas, we can formulate the expression for the power of the process of producing entropy:

(11) $$P_{diss}=T\;\Pi_{S}.$$

This is a case of thermal power of a caused process, which, in engineering, is called *dissipation rate.* (Section 4.4 in [2]; [3,4,5,6,7,8,9,10,11,12]).

For chemical reactions, having the notions of chemical potential difference as *chemical drive* for the reaction available makes dealing with such processes rather intuitive [2,18,19,44]. First, we can state explicitly what we mean by a *spontaneous chemical reaction:* it is one for which the chemical potential difference (chemical driving force) is negative (i.e., where the reaction is running “downhill”). Moreover, in this case, the reaction makes energy available. Second, we can suggest how the power of a spontaneous reaction such as the one described by Equation (4), i.e., the rate at which energy is made available, must depend upon the individual production/destruction rates—and therefore also on the overall reaction rate. When $n_{Ai}$ moles of a substance $A_{i}$ is consumed (disappear; are destroyed), this will make an amount of energy equal to $n_{Ai}\mu_{Ai}$ available. Alternatively, if $n_{Bi}$ moles of a substance $B_{i}$ is produced, this will “consume” $n_{Bi}\mu_{Bi}$ units of energy. Using general algebraic notation, with $\nu_{i}<0$ and $\nu_{i}>0$ for educts and products, respectively, we obtain

(12) $$∆\mu_{R}=\sum \nu_{i}\mu_{i}$$

for the *overall chemical potential difference* for the reaction. This leads to

(13) $$P_{ch}=∆\mu_{R}\Pi_{nR},$$

for the power of a chemical reaction. Note that $∆\mu_{R}<0$ for a spontaneous reaction, which means that P<0 for a spontaneous process (which holds generally for all spontaneous processes, as they make energy available). 

## 3. A Model of Exothermic and Endothermic Chemical Reactions

Basing our reasoning upon the fundamental quantities and relations introduced in the foregoing section makes modeling and understanding of the thermal balance of chemical reactions rather easy. Here, we shall construct the model and derive the relations that let us understand when reactions are either exothermic or endothermic. Put formally, we want to know if $I_{S,cond}<0$ or $I_{S,cond}>0$. The derivation of the model will be based upon the primitive concepts of chemical and thermal dynamics. 

### 3.1. Basing the Model upon the Primitive Concepts of Chemical and Thermal Processes

To obtain the equation for the conductive entropy flow due to heating/cooling—which will be our main result—we must first derive the relation for the entropy production rate in a reaction that is completely dissipative. This condition means that $P_{diss}=-P_{ch}$, which has already been suggested in Figure 1. Through Equation (11), we obtain

(14) $$\Pi_{S}=-∆\mu_{R}\Pi_{nR}/T,$$

for the entropy production rate (which will be a positive quantity: $∆\mu_{R}$, as a driving force, is negative, and $\Pi_{nR}$ has been taken as positive throughout).

Next, we use the dynamical form of the law of balance of entropy, as in Equation (2). If we insert the expressions for the convective entropy current and the production rate of entropy given in Equations (7) and (14), respectively, and observe that $dS/dt=d\left(\sum {\hat{s}}_{i}n_{i}\right)/dt$, and then use the product rule, we have

(15) $$\sum {\hat{s}}_{i}dn_{i}/dt+\sum n_{i}d{\hat{s}}_{i}/dt=I_{S,cond}+\sum {\hat{s}}_{i}I_{ni}-∆\mu_{R}\Pi_{nR}/T.$$

If we use Equation (1) for replacing $I_{ni}$ and define the change of molar entropy of the reaction from educts to products as

(16) $$∆{\hat{s}}_{R}=\sum \nu_{i}{\hat{s}}_{i},$$

we obtain

(17) $$I_{S,cond}=\sum n_{i}d{\hat{s}}_{i}/dt+\left[∆\mu_{R}/T+∆{\hat{s}}_{R}\right]\Pi_{nR}.$$

Again, we must point out that $\Pi_{nR}>0$, $∆\mu_{R}<0$, and $∆{\hat{s}}_{R}$ will be positive if the products carry more entropy (have a larger molar entropy) than the educts and will be negative if the products carry less entropy than the educts. This result holds for special cases as well, such as steady-state flow (plus reaction), batch reactions without any flows, and reactions where only some of the substances flow (such as when carbon dioxide escapes from a container where citric acid and baking soda in aqueous solution react, and the solution becomes cold). 

If we are interested in the energy current flowing as a consequence of heating/cooling, we can simply multiply Equation (17) by the temperature T (see Equation (9)). The quantity of energy communicated during a period—which is called heat in traditional equilibrium thermodynamics—can be obtained by integrating the expression thus obtained; see Section 5.2 for details.

### 3.2. Interpreting the Conditions for Heating/Cooling

**Figure 3:**
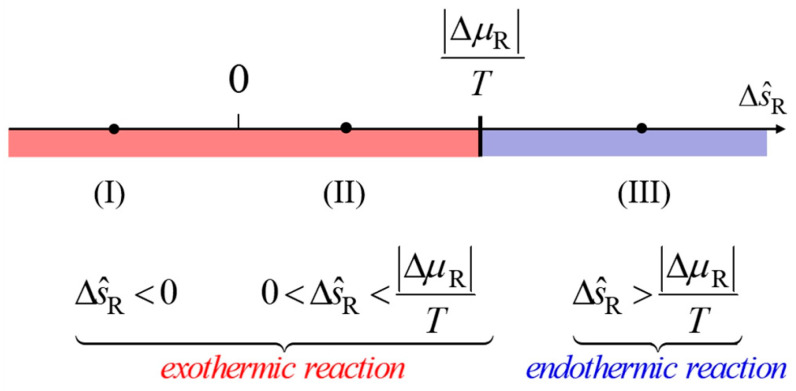
Analysis of the different cases relevant for the thermal balance of a reaction, as discussed here: in the case of a steady-state reaction, the aspects of the figure remain unchanged over time; in the case of equilibrium being approached, as the process progresses, the dividing line between exothermic and endothermic regimes gradually shifts toward zero ($∆\mu_{R}\to 0$).

We can now say what makes an endothermic reaction and what makes an exothermic one (see Figure 3). Note that the first term on the right-hand side of Equation (17) equals the rate of change of entropy of the substances momentarily in the reactor—formally, it is the sum of the products of rate of change of molar entropy (for simple fluids, as a consequence of changes of pressure and temperature) and amount of substance of all the species taking part in the reaction.

If we want to understand (and calculate) as easily as possible what makes a reaction exothermic or endothermic, it helps to restrict the model to steady-state operation, as depicted in Figure 1. This means that the first term on the right-hand side of Equation (17) can be neglected, and we obtain

(18) $$I_{S,cond}=\left[∆\mu_{R}/T+∆{\hat{s}}_{R}\right]\Pi_{nR}.$$

Since the first term in this expression will always be negative, we can obtain a positive conductive entropy current—i.e., an endothermic reaction—only if $∆{\hat{s}}_{R}$ is sufficiently greater than zero (so that $∆{\hat{s}}_{R}>\left\lceil ∆\mu_{R}/T\right\rceil$; see Figure 3). This simply means that the amount of entropy produced is not enough to compensate for the extra amount flowing out with the products. 

In general, Equation (18) suggests that we should expect three cases for spontaneous reactions. Indeed, in the expression for the conductive current there are two terms: the first is related to the production of entropy, and the second is due to the difference of entropy between reactants and products. As noted above, the latter can take on both positive and negative values, so that depending upon the relative weights of the two terms, we are faced with three different situations, as shown in Figure 3.

## 4. Examples of Spontaneous Steady-State Exothermic and Endothermic Reactions

Based upon the foregoing analysis, we are ready to present quantitative examples for each of the three cases of spontaneous reactions shown in Figure 3. Temperature and pressure conditions are assumed to be the reference conditions (25.0 °C, 1.01 bar), and values for chemical potentials and molar entropies can be obtained from the tables given in [45], where molar entropy is the negative temperature coefficient of the chemical potential. We further assume that we have a reactor operating at steady-state, and we choose a reaction rate of $\Pi_{nR}=1.0\times 10^{-3}$ mol/s. In all three examples, the chemical drive is negative, and the reactions are spontaneous. Note that we are discussing the case of steady-state operation (Equation (18)). We will say just a few words about how to deal with dynamical reactions at the end of Section 5.

### 4.1. Example I: Methane Combustion—Exothermic Process

Consider, for example, the combustion of methane: Table 1a shows the values for the molar entropies and chemical potentials of the different chemical species involved in the reaction (for standard conditions), while Table 1b shows the numerical values of the three terms relating to entropy as they appear in Equation (18) and Figure 3 (corresponding to the assumed of reaction rate of $\Pi_{nR}=1.0\times 10^{-3}$ mol/s, as mentioned above).

We can see that both contributions that determine the intensity of the conductive entropy exchange with the surroundings are negative; this means that there is a net flow from the system to the surroundings, i.e., we are dealing here with an exothermic reaction.

### 4.2. Example II: Decomposition of Hydrogen Peroxide—Exothermic Process

As an example of the second type of reaction, let us consider the reaction of decomposition of hydrogen peroxide. Table 2a shows the values for the molar entropies and chemical potentials of the different chemical species involved in the reaction (for standard conditions), while Table 2b shows the numerical values of the three terms appearing in the balance equation for entropy, Equation (18) (again corresponding to a reaction rate of $\Pi_{nR}=1.0\times 10^{-3}$ mol/s).

This time, we can observe that the term related to the change of molar entropy is positive; this means that, in a steady-state process, the convective outflow of entropy with products is greater than the corresponding inflow with reactants. However, the rate of entropy production is greater than this value; this means that, to maintain the steady-state at constant temperature, there must be a net flow of entropy from the reactor to its surroundings, i.e., the reaction is still exothermic.

### 4.3. Example III: Reaction of Barium Hydroxide and Ammonium Nitrate—Endothermic Process

As an example of the third group, let us consider the reaction between barium hydroxide and ammonium nitrate: the large difference (in favor of the products) in molar entropy (see Table 3a) means that in order to maintain the steady-state at constant temperature, it is necessary to conductively import entropy from the surroundings ($I_{S,cond}>0$). Thus, the reaction turns out to be endothermic (Table 3b).

## 5. Some Didactic Considerations

Clearly, the approach to chemical and thermal dynamics based upon experiential primitives differs from the traditional method. At least two issues arise that we would like to discuss: (1) how to prepare learners to deal efficiently with the direct imaginative approach presented here (what are possible prerequisites to theories of chemical and thermal dynamics?), and (2) what does it take to connect the results obtained by our direct approach to traditional indirectly derived concepts (if and when students are subsequently exposed to a traditional presentation of the subject)?

After discussing these issues, we shall present arguments that let us prefer the direct approach to experiential primitives and relations as applied to dynamical processes developed here.

### 5.1. Preparing Learners for a Direct Approach to Thermal and Chemical Dynamics

The answer to the first question is simple: we need to expose learners to a couple of subjects where experiential primitives arise most directly, where creating dynamical models is simple, and where the power of analogical reasoning can be demonstrated most clearly. Here, we quickly sketch the typical structure of an introductory course that we have created and taught—in various forms suited to different audiences—for several decades.

Look at the concepts presented in Section 2—the question is, how do we arrive at an understanding and mastery of what has been outlined there in the most superficial and brief manner? Phenomena related to the flow of fluids at the surface of the Earth offer the clearest path to what we need (we treat incompressible fluids, if we want to create a particularly simple first encounter with the issues; (Chapter 1 in [2])). We learn about the (dynamical, steady-state, and integrated) law of balance of a conserved quantity (volume of fluid and mass of fluid), and about the role of vertical level and pressure differences as the drive for hydraulic and gravitational processes (combining these aspects allows us to study and formulate constitutive relations found in capacitive, resistive, and inductive elements). The coupling of hydraulic processes to gravitational ones, i.e., when fluids flow vertically at the surface of our planet, introduces us for the first time to the notion of power of a process (the image for which we take from Carnot [20]; see (Chapter 2 in [2])), which we then extend to create a first encounter with the generalized energy principle.

While the use of analogy arises first when these couplings are introduced, analogical reasoning becomes most powerful and consciously experienced when the form of the models created in gravito-fluid phenomena is extended to a new field, which we usually take to be electrical circuits (Chapter 1 in [2])). Models are structured with the help of concepts (electric charge and its flow, electric potential differences, and the notion of power of an electric process when charge flows through an electric tension), and relations are formulated in analogy to what has been encountered before. 

Finally, learning to understand entropy as the extensive quantity of thermal phenomena—again in analogy to previously studied processes, now with temperature as the corresponding potential—is important for direct experiential access to a dynamical theory of heat. The equation of balance of entropy (as in Equation (2)) establishes the connection with irreversibility. Furthermore, Carnot’s principle gives us the tool needed for formulating the relation between flows of entropy, thermal tension, and thermal power; this leads to simple imaginative models of heat engines. Process diagrams (as in Figure 3 of [3]) express this knowledge in concrete form and introduce students in a graphically visual manner to the distinction between first- and second-law efficiencies. If we structure an introduction to thermal phenomena along these lines (Chapters 4–6 in [2]; Chapter 4 in [46])), we are ready for the treatment of chemical processes and thermochemical coupling as presented here in Section 2 and Section 3.

### 5.2. Energy Exchanged in Heating/Cooling and Thermodynamic Potentials

Let us now show how the traditional energy-related concepts can be derived from the work presented above. We start from our main result, formulated in Equation (17)—restricted to steady-state processes, as in Equation (18). Taking Equation (9) for an energy current in heating/cooling, we obtain

(19) $$I_{E,th}=\left[∆\mu_{R}+T∆{\hat{s}}_{R}\right]\Pi_{nR}.$$

This is the energy current associated with heating/cooling of what is in the reactor. The integral over time of the expression on the left-hand side is what is called (quantity of) heat Q in traditional thermodynamics:

(20) $$Q=\int TI_{S,cond}dt.$$

This brings us to the issue of *thermodynamic potentials*, i.e., enthalpy and Gibbs free energy, which are purely formal auxiliary constructs. For the following definitions and relations to hold, we need to assume conditions of fixed pressure and temperature of a fluid mixture of chemicals. 

Enthalpy is defined as H=E+pV; while all three variables on the right-hand side have figurative meaning, their combination forming H does not—it is but a convenient abbreviation of terms occurring in some energy balances. However, under special conditions, we can establish a relation between H and the energy exchanged in heating/cooling. We start with the generic balance of energy (as in Equation (3)) for a body of fluid in a form containing energy transferred as a consequence of (1) heating/cooling ($I_{E1}=I_{E,th}$) and (2) volume change of the fluid characterized by pressure p ($I_{E2}=-pdV/dt$). Using the definition of enthalpy, we obtain $TI_{S,cond}=dH/dt-Vdp/dt$. If we accept p=const. and integrate over time, we have

(21) ∆H=Q.

Note that this relation holds for thermofluidic processes whether or not reactions take place. Second, we formally introduce the integrals on the right-hand side of Equation (19). The first part is called the change of the Gibbs free energy:

(22) $$\Delta G_{R}=\int ∆\mu_{R}\Pi_{nR}dt,$$

whereas the second part is left “undefined” and simply equals

(23) $$T\Delta S_{R}=\int T∆{\hat{s}}_{R}\Pi_{nR}dt,$$

for the stated conditions of isobaric and isothermal operations. In sum, we obtain the well-known Gibbs–Helmholtz equation for $∆G_{R}$:

(24) $$\Delta G_{R}=\Delta H_{R}-T\Delta S_{R}.$$

In traditional chemical thermodynamics, it is customary to tabulate values of Gibbs free energy and enthalpy for standard conditions, rather than chemical potentials and (molar) entropies; see, for instance, [47,48]. Naturally, we obtain the values reported in such tables if we use our direct approach to chemical and thermal dynamics. Results for our numerical examples reported in Table 1, Table 2 and Table 3 are given in Table 4, calculated for a steady-state reaction where the total chemical amount involved (referring to the stoichiometry) is exactly $n_{R}=$ 1.0 mol.

Remember that $∆H_{R}^{0}$ equals the energy exchanged together with entropy, i.e., what is commonly called “heat” exchanged; it is calculated from the entropy exchanged with the help of Equation (20). Take the example of methane in Table 1. According to the rates assumed, 1 mol of methane will have reacted in 1000 s. This means that the amount of entropy exchanged during this period will be equal to –2.99 kW/K. Multiplied by 298.15 K, we obtain the amount of energy exchanged, as reported in Table 4.

### 5.3. The Power of a Direct Approach to Experiential Primitives

Let us discuss what we see as important advantages of the approach presented in this paper. Above all, the use of chemical potential, amount of substance, temperature, and entropy as primary quantities allows learners and practitioners to imaginatively and figuratively understand the concepts that they use [3], and work with a coherent picture within which they could recognize structural similarities (i.e., analogies in the sense of structure mapping; see [2,13,16,36]) in the description of the various fields of macroscopic physical science [2,8,9,10,11,12]. This leads to great economy in learning about and modeling complex dynamical systems and processes.

One of the important contributions of this paper deals with the immediate use of chemical potential and entropy—for which we possess simple and clear imagery—as primitives. This makes it possible to present (a) spontaneity analysis and (b) thermal balance considerations in a straightforward manner. Not only does the chemical potential unify our understanding of chemical phenomena [18,19,33], it directly tells us what makes a spontaneous reaction and what we mean by the power of a chemical process, and mastering a direct entropic approach to thermal processes explains—without recourse to derived concepts such as thermodynamic potentials, for which imagery is lacking—how a reaction is either exothermic or endothermic.

Not taking recourse to the primitive concepts of thermal and chemical dynamics, as is the case in traditional approaches to physical chemistry, is one of the sources for needing classical thermodynamic potentials—we burden the energy concept with what can only be understood through models making use of the fundamental concepts and relations of the phenomena involved in a particular system and its processes. For example, for spontaneity of processes at constant temperature and pressure, Gibbs free energy G is often used, while for thermal balances, again under the same conditions, enthalpy H is used. These two quantities are then related by the Gibbs–Helmholtz equation ΔG=ΔH−TΔS. This reminds us of building and using crutches when walking freely is entirely possible and clearly desirable.

Compare our means for understanding chemical potential vs. Gibbs free energy. The former uses a class of concepts—namely, intensities and their differences, i.e., tensions—to which we have direct experiential access [2,3,14,33,46]. Contrast this with G=E+pV−TS. We may understand the meaning of pressure, volume, and temperature and, if we are generous, even of energy. Now, even if we understood entropy as traditionally introduced, there is no way we can form an image for the combination of terms appearing in the definition of G. No wonder we are hobbled in our desire to master important thermochemical theories and models—research has provided us with ample evidence of this deplorable situation [24,25,26,27,28,29,30,31,32,49,50,51]. It may perhaps be interesting in this regard to cite the historical note by Prigogine (Section 4.1 in [52]) referring to the establishment of the habit of using the term Gibbs free energy in place of the affinity $A=-\Delta \mu_{R}$, which was taken up and refined by De Donder’s school [53]. Striving for coherent experiential imagery and conceptualization, we find the widespread use in textbooks of the concept of Gibbs free energy (which is an extensive quantity!) for the characterization of the notions of drive and equilibrium particularly problematic; this is a choice that obviously hides some important aspects. Only a few textbooks bring into proper focus the fact that the equilibrium condition is related to the stationarity of the free energy when it is represented as a function of the degree of progress of the reaction: This is done graphically (horizontal tangent) or more formally by making use of Lewis’ operator. In fact, as with all other areas, equilibrium is a situation in which “levels” take the same values: electrical potential for electrical phenomena, temperature for thermal phenomena, etc.; the explicit use of chemical potential avoids this conceptual obstacle.

Regarding entropy, we have discussed the experiential aspects of this concept as a primitive (along with temperature) in [3]. Our approach presented here is based upon quantities that can be effectively introduced at a phenomenological–macroscopic level [1,2,3,4,5,6,7,8,9,10,11,12]; this avoids the dangerous mixing of the macroscopic and microscopic approaches, which is unavoidable in the traditional approach, in which the entropic aspect is supported by (usually) unspecified order/disorder criteria [29,54].

### 5.4. The Power of a Dynamical Systems Approach to Processes

There is a second aspect that sharply distinguishes between traditional approaches and the one developed here. Traditional treatments are based upon (quasi-static) equilibrium theories of thermal and chemical processes. While we may read of “processes for which pressure and temperature are kept constant”, there are no processes but only initial and final states for which pressure and temperature are the same, and to which changes of Gibbs free energy and enthalpy are then applied—we will never know what happened between beginning and end. Furthermore, it is not possible to relate entropy production to (quasi-static) reversible processes. Prigogine and Kondepudi [52] (p. 111) write: “We will consider explicit examples of entropy production due to chemical reactions […] [A]ffinity is a concept that relates irreversible chemical reactions to entropy, whereas Gibbs free energy is primarily used in connection with equilibrium states and reversible processes. Nevertheless, in many texts the Gibbs free energy is used in place of affinity and no mention is made of the relation between entropy and reaction rates.” Put simply, time does not exist in equilibrium theories of thermal and chemical phenomena, and neither do initial value problems in ordinary or partial differential equations. This is in stark contrast to what we obtain and understand if we take the route outlined by CP and UDS.

For example, if we deal with a system that starts in a non-equilibrium state and from there evolves towards equilibrium, we need to develop dynamical models. Starting, as we have done, with the dynamical form of laws of balance, as in Equations (1)–(3), we can create full-blown dynamical models in UDS by joining the balance relations with proper constitutive relations. In this regard, it is useful to remember that the description of the real dynamics of the chemical process requires modeling based on knowledge of the kinetics peculiar to the specific process: in some cases it can be observed experimentally and conveniently modeled (as in the case of D-glucose mutarotation [17,18]). In many cases, the rapidity of the chemical process and its “causes” proceed on different time scales (such as in a titration process—given the quickness of the acid-base neutralization reaction—it is essentially the rapidity with which the reagent is introduced that determines the temporal evolution of the pH of the system). In this case, dynamic modeling allows the temporal evolution of the process to be followed. The use of appropriate graphs showing the degree of reaction progress also allows students to view a kind of animation of the process (the examples in [55] can be taken up and effectively presented to students with Geogebra). Beyond what we have presented here, we will need to make use of a kinetic equation for the reaction and add expressions for the molar entropy of disappearing and appearing substances, thus creating a system of initial value problems for the types of phenomena that we have discussed here (Chapter 6 in [2]). We shall present a concrete example of experiments and dynamical models in a future paper.

## 6. Conclusions

Basing the thermal balance of a chemical reaction explicitly upon chemical potential and the balance of entropy offers numerous advantages from conceptual, educational, and practical perspectives. In fact, it allows for an experiential path to the conceptualization of the spontaneity of a reaction, and for a clear distinction to be made between two otherwise indistinguishable contributions to the “heat” balance: the first due to the difference in entropy between reactants and products, commonly associated with latent heat (which can take on both positive and negative values), and the second resulting from the production of entropy associated with a totally dissipative process (which can take on only positive values). The latter aspect can be easily highlighted by the notion of chemical power, i.e., by using the concept of chemical potential and its differences; all of the energy made available by a spontaneous chemical process, i.e., one characterized by a negative chemical potential difference between reactants and products, is dissipated (i.e., entropy is produced). The sum of these two contributions then defines the overall thermal balance and makes it possible to determine whether a given chemical reaction can be used as a heating or cooling source, and whether and how a given chemical reactor must be cooled or heated.

Moreover, note that the approach presented here makes it possible to free the discussion of the spontaneity of a chemical reaction from entropic and/or energetic considerations; in fact, it suffices to consider changes in chemical potential for this purpose. Finally, our analysis has general validity and is not limited to processes occurring at constant pressure and temperature, as is the case if changes of thermodynamic potentials are considered.

Finally, the generalized energy principle developed in continuum physics and the physics of uniform dynamical systems makes energy into a sharp and precise tool for what and where it is needed—essentially, for explaining and quantifying the coupling of processes—rather than a jack of all trades that cannot possibly satisfy all of the demands with which it has been saddled traditionally. Put simply, the concept of energy does not have the explanatory power we might wish to associate with it—overextending its use hides what is happening in processes.

## Figures and Tables

**Table 1:** Methane combustion.

	CH_4_ + 2O_2_	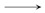	CO_2_ + 2H_2_O			
$\begin{array}{c} {\hat{s}}^{0}\\ {}[J/(K\;\mathrm{mol})] \end{array}$	$\underset{596}{\underset{︸}{186\;+2\times 205}}\;$		$\underset{351}{\underset{︸}{213\;+2\times 69}}\;$		$\begin{array}{c} \Delta {\hat{s}}_{R}^{0}\;\pi_{n_{R}}\\ \left[W/K\right] \end{array}$	−0.25
$\begin{array}{c} \mu^{0}\\ \left[\mathrm{kJ}/\mathrm{mol}\right] \end{array}$	$\underset{-50}{\underset{︸}{-50\;+2\times 0}}\;$		$\underset{-868}{\underset{︸}{-394\;+2\times (-237)}}$		$\begin{array}{c} \Delta \mu_{R}^{0}\;\pi_{n_{R}}/T^{0}\\ \left[W/K\right] \end{array}$	−2.74
					$\begin{array}{c} I_{S\;cond}\\ \left[W/K\right] \end{array}$	−2.99
(a)					(b)	

**Table 2:** Decomposition of hydrogen peroxide.

	2 H_2_O_2_	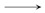	2 H_2_O + O_2_			
$\begin{array}{c} {\hat{s}}^{0}\\ {}[J/(K\;\mathrm{mol})] \end{array}$	$\underset{219.2}{\underset{︸}{\;2\times 109.6\;}}\;$		$\underset{344.8}{\underset{︸}{\;2\times 69.9\;+\;205}}\;$		$\begin{array}{c} \Delta {\hat{s}}_{R}^{0}\;\pi_{n_{R}}\\ \left[W/K\right] \end{array}$	+0.125
$\begin{array}{c} \mu^{0}\\ \left[\mathrm{kJ}/\mathrm{mol}\right] \end{array}$	$\underset{-240.8}{\underset{︸}{\;2\times (-120.4)}}\;$		$\underset{-474.4}{\underset{︸}{\;2\times (-237.2)+\;0}}\;$		$\begin{array}{c} \Delta \mu_{R}^{0}\;\pi_{n_{R}}/T^{0}\\ \left[W/K\right] \end{array}$	−0.784
					$\begin{array}{c} I_{S\;cond}\\ \left[W/K\right] \end{array}$	−0.659
(a)					(b)	

**Table 3:** Reaction between barium hydroxide and ammonium nitrate.

	Ba(OH)_2_·8H_2_O + 2NH_4_NO_3_	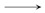	2NH_3_ + 10 H_2_O + Ba(NO_3_)_2_			
$\begin{array}{c} {\hat{s}}^{0}\\ {}[J/(K\;\mathrm{mol})] \end{array}$	$\underset{729.0}{\underset{︸}{426.8\;+\;2\times 151.1}}\;$		$\underset{1297.8}{\underset{︸}{\;2\times 192+10\times 70+213.8}}\;$		$\begin{array}{c} \Delta {\hat{s}}_{R}^{0}\;\pi_{n_{R}}\\ \left[W/K\right] \end{array}$	+0.57
$\begin{array}{c} \mu^{0}\\ \left[\mathrm{kJ}/\mathrm{mol}\right] \end{array}$	$\underset{-3161}{\underset{︸}{\;-2793\;+\;2\times (-184)}}\;$		$\underset{-3197}{\underset{︸}{\;2\times (-16)+10\times (-237)+(-795)}}\;$		$\begin{array}{c} \Delta \mu_{R}^{0}\;\pi_{n_{R}}/T^{0}\\ \left[W/K\right] \end{array}$	−0.12
					$\begin{array}{c} I_{S\;cond}\\ \left[W/K\right] \end{array}$	+0.45
(a)					(b)	

**Table 4:** Comparison of the three kinds of reactions discussed in Table 1, Table 2 and Table 3.

		$\begin{array}{c} T^{0}\;\Delta S_{R}^{0}\\ \left[\mathrm{kJ}\right] \end{array}$	$\begin{array}{c} \Delta G_{R}^{0}\\ \left[\mathrm{kJ}\right] \end{array}$	$\begin{array}{c} \Delta H_{R}^{0}\\ \left[\mathrm{kJ}\right] \end{array}$	
Example I	*Combustion of methane*	−73.1	−818.0	−891.1 < 0	*Exothermic reaction*
Example II	*Decomposition of hydrogen* *peroxide*	37.4	−233.8	−196.4 < 0	*Exothermic reaction*
Example III	*Reaction between barium* *hydroxide and ammonium nitrate*	169.4	−36.0	133.4 > 0	*Endothermic reaction*

## Data Availability

No new data were created or analyzed in this study. Data sharing is not applicable to this article.
